# Ira Hatch, M. D.

**Published:** 1879-11

**Authors:** 


### Obituary.

### IRA HATCH, M.D.

Resolutions adopted by the Chicago Medical Society at a meeting held October 20, 1879:

WHEREAS, Our beloved friend and brother, Ira Hatch, M.D., has by death been removed from his sphere of usefulness, therefore,

*Resolved*, That in his death we, as a society, have sustained a loss that words are inadequate to express.

*Resolved*, That our deceased brother was valued by his colleagues, and by all who knew him, as a thoroughly truthful, straightforward man, a man of excellent judgment, a keen observer and successful practitioner. He has always been an active member of our society; had presented several papers containing valuable practical information; and though at an advanced age, always, to the last, retained an interest in the advancement of medical science.

*Resolved*, That we tender the afflicted family our sympathies in this sad hour of their bereavement.

*Resolved*, That the secretary furnish a copy of these resolutions to the family of the deceased. Also a copy to each of the papers of this place, and to the CHICAGO MEDICAL JOURNAL AND EXAMINER.

(Signed,) G. C. PAOLI, M.D.,

EDWIN POWELL, M.D.,

B. MCVICKAR, M.D.,

} *Committee.*

